# *RgC3H* Involves in the Biosynthesis of Allelopathic Phenolic Acids and Alters Their Release Amount in *Rehmannia glutinosa* Roots

**DOI:** 10.3390/plants9050567

**Published:** 2020-04-29

**Authors:** Yanhui Yang, Zhongyi Zhang, Ruifang Li, Yanjie Yi, Heng Yang, Chaojie Wang, Zushiqi Wang, Yunyi Liu

**Affiliations:** 1 College of Bioengineering, Henan University of Technology, Lianhua Street 100, Zhengzhou High-Technology Zero, Zhengzhou 450001, China; lrf@haut.edu.cn (R.L.); yiyanjie@haut.edu.cn (Y.Y.); 201893188@stu.haut.edu.cn (H.Y.); 201992163@stu.haut.edu.cn (C.W.); 201812030215@stu.haut.edu.cn (Z.W.); 201812030330@stu.haut.edu.cn (Y.L.); 2 College of Crop Sciences, Fujian Agriculture and Forestry University, Jinshan Road, Cangshan District, Fuzhou 350002, China; zyzhang@fafu.edu.cn

**Keywords:** *R. glutinosa*, allelopathic compounds, root exudates, *p*-Coumarate-3-hydroxylase, Phenolic acid biosynthesis, release amount

## Abstract

*Rehmannia glutinosa* production is affected by replanting disease, in which autotoxic harm to plants is mediated by endogenous phenolic acids as allelopathic compounds found in root exudates. These phenolic acids are mostly phenylpropanoid products of plants’ secondary metabolisms. The molecular mechanism of their biosynthesis and release has not been explored in *R. glutinosa*. *P*-coumarate-3-hydroxylase (C3H) is the second hydroxylase gene involved in the phenolic acid/phenylpropanoid biosynthesis pathways. C3Hs have been functionally characterized in several plants. However, limited information is available on the *C3H* gene in *R. glutinosa*. Here, we identified a putative *RgC3H* gene and predicted its potential function by in silico analysis and subcellular localization. Overexpression or repression of *RgC3H* in the transgenic *R. glutinosa* roots indicated that the gene was involved in allelopathic phenolic biosynthesis. Moreover, we found that these phenolic acid release amount of the transgenic *R. glutinosa* roots were altered, implying that *RgC3H* positively promotes their release via the molecular networks of the activated phenolic acid/phenylpropanoid pathways. This study revealed that *RgC3H* plays roles in the biosynthesis and release of allelopathic phenolic acids in *R. glutinosa* roots, laying a basis for further clarifying the molecular mechanism of the replanting disease development.

## 1. Introduction

*R. glutinosa*, a species of the *Scrophulariaceae* family, is a perennial herbaceous plant. Its tuberous roots contain a number of pharmacologically active compounds and are used in traditional Chinese medicine [1,2]. However, this medicinal plant is affected by replanting disease, in which the continuous monoculture of *R. glutinosa* leads to serious negative effects on both plant health and the rhizosphere soil, resulting in a marked decline in the biomass and quality of underground tuber roots [3,4,5]. Root exudates are perceived as chemical signals of communication between roots and microorganisms [6,7,8]. A growing body of evidence suggests that autotoxic harm to plants mediated by root exudates plays a crucial role in replanting disease [3,6,9,10]. Some endogenous phenolic acids, which are important root exudates, are involved in allelopathy and are prone to directly or indirectly cause autotoxicity, resulting in replanting disease in some plants [6,11,12,13]. Several phenolic acids (e.g., ferulic acid (FA), caffeic acid (CA) and chlorogenic acid (CHA)) have been identified as allelopathic autotoxic agents in *R. glutinosa* and other plants [3,6,13]. These phenolic acids are mostly phenylpropanoid products of secondary metabolism in plants [3,13,14,15]. A thorough understanding of the biosynthesis and release of these allelopathic phenolic acids and the characterization of the enzymes involved are important for elucidating the underlying mechanisms of the development of replanting disease, especially in the case of the production of the medicinal plant parts (roots) of *R. glutinosa*. 

Many of the general phenylpropanoid pathways for phenolic acid biosynthesis require at least two hydroxylases in plants [15,16]. *P-*coumarate-3-hydroxylase (C3H), which is a cytochrome P450 monooxygenase belonging to the CYP98A family (CYP98A3) [17], is the second hydroxylase in the biosynthesis pathways of some allelopathic phenolic acids, such as FA, CA, and CHA [18,19,20]. Phenolic acid biosynthesis via C3H catalyzation in plants starts with either L-phenylalanine or L-tyrosine [18,21] (Figure S1). L-phenylalanine is deaminated by phenylalanine ammonia-lyase (PAL) to form transcinnamic acid, and a hydroxyl group is then added to the 4-position of the aromatic ring by cinnamate 4-hydroxylase (C4H) to form *p*-coumarate. *P*-coumaric acid can also be synthesized through the direct deamination of tyrosine by tyrosine ammonia-lyase (TAL). Then, the production of CA from *p*-coumarate as the *p*-coumarate-3-hydroxylase substrate requires *p*-coumarate-3-hydroxylase (C3H), which transfers another hydroxyl group to the 3-position of the aromatic ring of *p*-coumarate. Finally, the production of FA from CA requires caffeic acid O-methyltransferase (COMT), which catalyzes the O-methylation of CA to FA. FA provides the raw material that is eventually converted into its derivatives and other phenolic acids. *P*-coumarate can also be used for the synthesis of the thioester *p*-coumaroyl-CoA by 4-coumarate-CoA ligase (4CL), and *p*-coumaroyl-CoA can be transformed into *p*-coumaroyl-quininc acid, which is catalyzed by hydroxycinnamoyl-CoA quinate hydroxycinnamoyl transferase (CQT). Finally, the production of CHA from *p*-coumaroyl-quininc acid requires C3H hydroxylation, and CHA provides the raw material that is eventually converted into its derivatives and other phenolic acids [18,19,20,21,22].

C3Hs have been isolated and functionally characterized in some plants and have been shown to mediate plant growth and development and various stress responses via their involvement in the biosynthesis of many phenolics [15,16,18]. However, limited information about the role of C3Hs in the biosynthesis of these allelopathic phenolic acids is available in *R. glutinosa*. The molecular isolation and functional characterization of the *C3H* genes of these species are therefore important steps for illuminating these phenolic acid biosynthesis pathways. Here, a sequence from *R. glutinosa* was exploited to identify a putative *RgC3H* gene, whose full opening-read frame (ORF) sequence was isolated and predicted to encode a putative functional RgC3H protein. The subcellular localization of RgC3H was analyzed, and its exact function in the biosynthesis of FA, CA and CHA, as allelopathic phenolic acid agents, was elucidated on the basis of its overexpression and repression in *R. glutinosa*. Importantly, we investigated into the release amount of *RgC3H*-transgenic roots and tested the molecular networks involved in the phenolic acid/phenylpropanoid pathways via the regulation of *RgC3H*, laying a basis for the clarification of the molecular mechanism responsible for replanting disease development.

## 2. Results

### 2.1. Cloning and Sequence Analysis of the Full-Length RgC3H Gene

According to *R. glutinosa* transcriptome data, a putative sequence was annotated and shown to possess high homology to C3Hs from other plant species; this sequence was named RgC4H. The complete cDNA sequence of 1732 bp in length was amplified using polymerase chain reaction (PCR) technology and obtained by sequencing. The *RgC3H* gene was submitted to the NCBI GenBank database (Assession number MT081381). It was a full ORF of 1530 bp and encoded 509 amino acids. The putative RgC3H protein exhibited a molecular weight of 57.91 kDa and an isoelectric point (pI) of 8.89 (Table S1). The instability index was calculated to be 31.11, classifying the protein as stable. According to SOPMA analysis, the secondary structure of RgC3H was predicted to be mainly composed of alpha helices (51.87%) interspersed with random coils (31.83%), extended strands (9.43%), and beta turns (6.88%) (Table S2). The prediction of transmembrane (TM) domains using TMHMM2.0 suggested that there was one putative TM segment in RgC3H N-terminal sequence (Figure S2).

**Figure 1:**
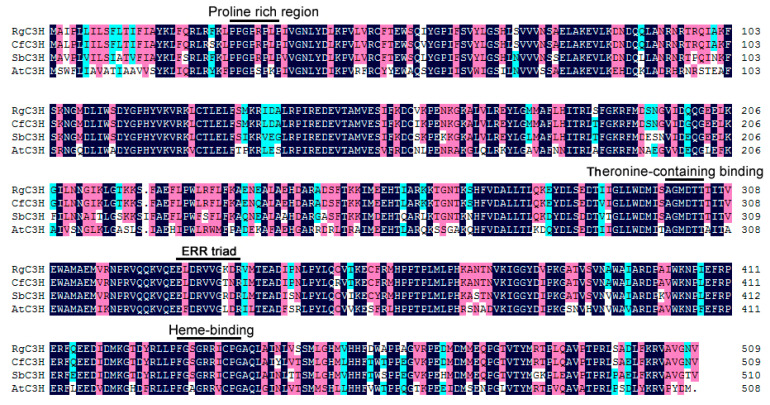
Alignment of the deduced RgC3H protein sequence with those of related C3Hs. Dark blue shading indicates amino acid identities, red and light blue shading indicates amino acids with differences in similarity. The locations of the putative conserved proline-rich region, heme-binding, threonine-containing binding pocket, and ERR triad motifs are shown above the sequences.

**Figure 2:**
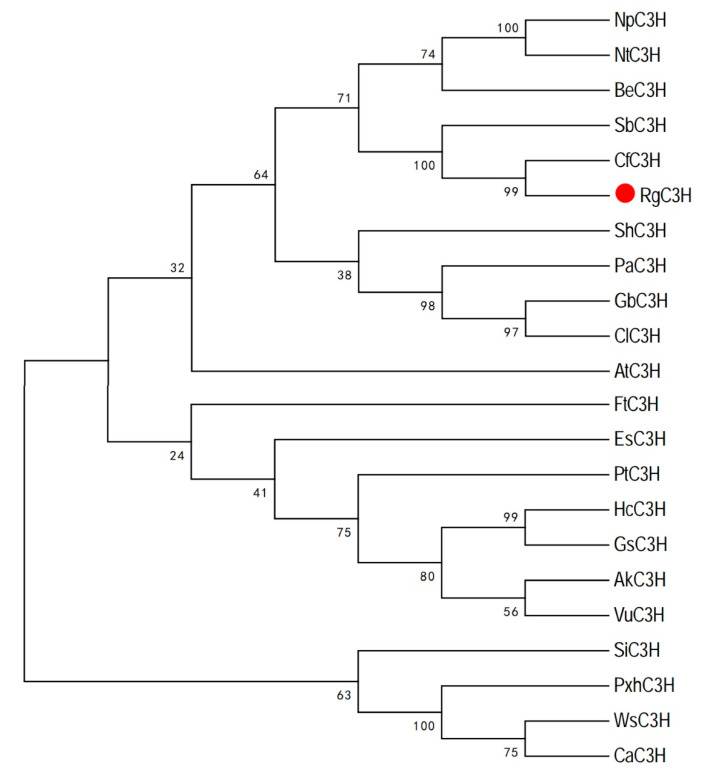
Phylogenetic tree of RgC3H and C3H sequences from other species.

Sequence analysis indicated that the RgC3H protein possessed a proline-rich region following the N-terminal anchoring sequence (P/IPGPXG/PXP), which is believed to be required for the protein’s correct orientation relative to the membrane. Similar to other P450s [15], the sequences included both a heme-iron ligand signature (PFGXGRRXCXG) near the C-terminal, a threonine-containing a binding pocket motif (A/GGXD/ETT/S) for the oxygen molecule required, and a ERR triad (EXXR...R) for catalysis (Figure 1). These results indicated that the RgC3H protein shares the typical characteristics of plant P450 proteins. Furthermore, amino acid sequence alignment revealed that the RgC3H protein shared 94.70%, 86.47% and 70.78% sequence identity with CfC3H (*Catalpa fargesii*), SbC3H (*Scutellaria baicalensis*) and AtC3H (*Arabidopsis thaliana*), respectively (Figure 1 and Table S2). A phylogenetic tree was constructed using the C3H amino acids from a variety of species to elucidate the evolutionary relationships among the C3Hs. RgC3H was located on a different branch of the tree from AtC3H and exhibited a close relationship with CfC3H (Figure 2).

### 2.2. Analysis of the Subcellular Localization of RgC3H

**Figure 3:**
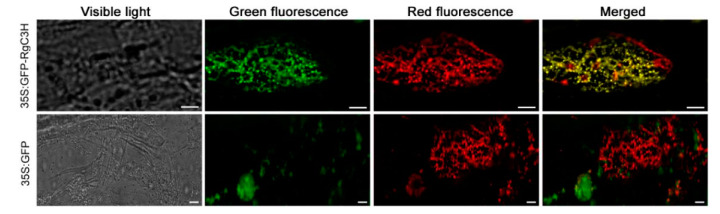
Subcellular localization of the CaMV35S:RgC3H-GFP fusion protein in onion epidermal cells. CaMV35S:GFP was used as a control, ER-Tracker (red fluorescence) was recruited to mark the ER, Bars = 50 µm.

Based on in silico localization analysis using the PSORT and Plant-mPLoc programs, RgC3H was predicted to be consistently located in the endoplasmic reticulum (ER) (Table S1). To experimentally determine the subcellular localization of RgC3H, the complete coding region of the gene was fused to the N-terminus of GFP (Figure S3). The CaMV35S:GFP-RgC3H construct was transiently expressed in onion epidermis. While both the green fluorescence from fusion protein of CaMV35S:GFP-RgC3H and the red fluorescence from ER Marker were detected by fluorescence microscopy, the merged yellow fluorescence from more than five transgenic cells was also observed and all of them had a similar result. One of them was presented here (Figure 3), confirming the in silico prediction of RgC3H ER localization.

### 2.3. Generation and Molecular Identification of Transgenic R. glutinosa

**Figure 4:**
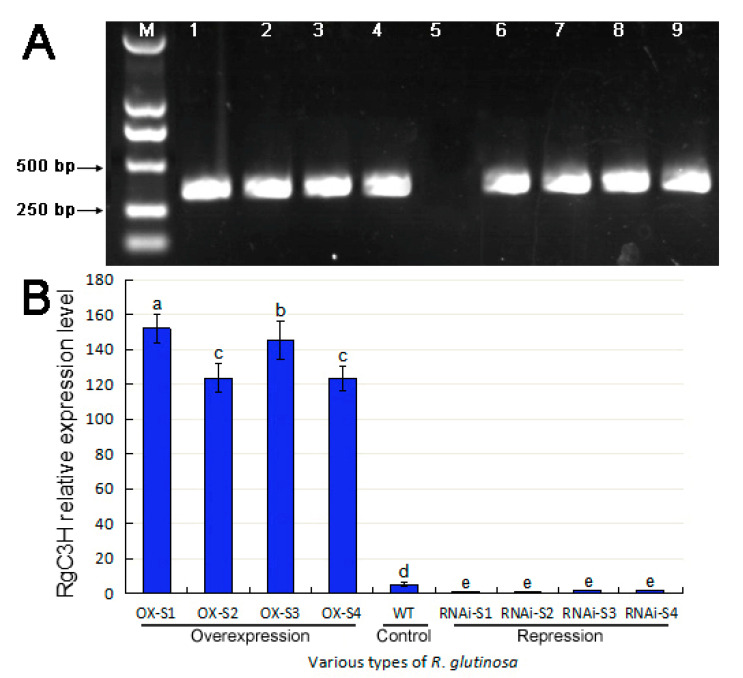
Confirmation of positive *RgC3H*-transgenic *R. glutinosa*. (**A**) PCR products for the positive screening of transgenic lines; Lanes M and 5 represent the DL2000 size marker and WT plants, respectively; Lanes 1–4 and 6–9 represent *RgC3H* overexpression and repression lines, respectively; (**B**) *RgC3H* expression patterns in these roots. The error bars represent the standard error (*n* = 3) (*p* < 0.05); the lowercase letters (a–e) represents different significant signs, the below is same.

To investigate the molecular function of the *RgC3H*, we constructed the *RgC3H* overexpression (RgC3H-OX) and repression (RgC3H-RNAi) vectors under the control of the CaMV35S promoter (Figure S3). Then, we expressed the RgC3H-OX and C3H-RNAi constructs in *R. glutinosa*. Healthy transgenic RgC3H-OX and RgC3H-RNAi *R. glutinosa* lines were generated, and these plants were propagated in vitro (Figure S4). Moreover, the positive transgenic lines were confirmed by PCR analysis using DNA extracted from their roots. As expected, a 344 bp band of the CaMV35S promoter-driven gene was specifically amplified from the DNA of the eight transgenic plants, while the non-transgenic seedlings (the WT, as a control) showed no amplification (Figure 4A). In addition, the relative expression level of *RgC3H* in the roots of these transgenic lines was analyzed via quantitative real-time PCR (qRT-PCR) (Figure 4B). Among the lines, four overexpression lines (RgC3H-OX-S1, S2, -S3, and -S4) presented higher expression of *RgC3H* than was found in the WT, while four repression lines (RgC3H-RNAi-S1, -S2, -S3, and -S4) presented lower expression levels of *RgC3H* than the WT plants. The transcripts level of *RgC3H* in these overexpression lines (RgC3H-OX-S1, -S2, -S3, and -S4) were approximately 30.38-, 23.12-, 27.24-, and 23.04-fold that in the WT, respectively, while the transcripts levels of *RgC3H* in the four RNAi repression lines (RgC3H-OX-S1, -S2, -S3, and -S4) were approximately 0.19-, 0.20-, 0.39-, and 0.34-fold that of the WT, respectively. The results showed higher expression of *RgC3H* in all transgenic overexpression lines and lower expression in the transgenic RNAi lines compared with WT plants. Furthermore, the transcription levels varied between the transgenic lines of the same type (overexpression or RNAi repression). For example, the expression levels in the transgenic overexpression lines were significantly different, with the highest relative expression being found in the RgC3H-OX-S1 line, followed by RgC3H-OX-S3. However, the differences in the expression levels between the transgenic RNAi lines were not significant; among these lines, expression was lowest in RgC3H-RNAi-S1, followed by RgC3H-RNAi-S2. To assess the function of *RgC3H* in *R. glutinosa*, we chose the two overexpression lines with the highest expression (RgC3H-OX-S1 and RgC3H-OX-S3) and the two RNAi repression lines with the lowest expression (RgC3H-RNAi-S1 and RgC3H-RNAi-S2) for further analysis.

### 2.4. Analysis of Allelopathic Phenolic Acid Production in Transgenic R. glutinosa Roots

**Figure 5:**
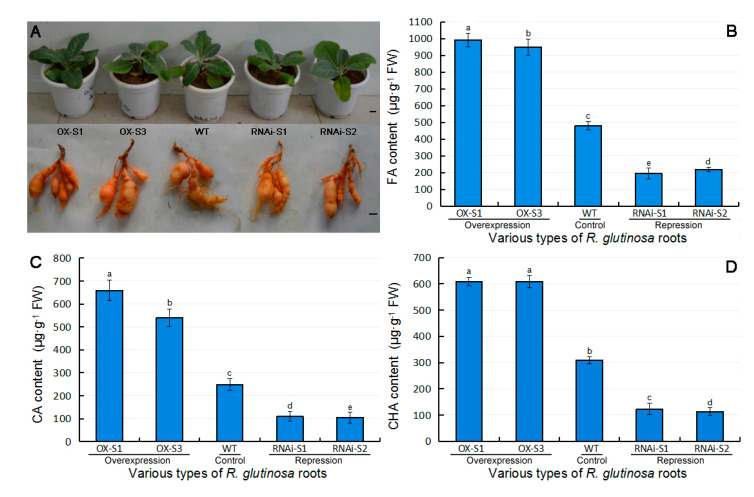
The morphology and accumulation of several phenolic acids in WT and *RgC3H*-transgenic *R. glutinosa* roots. (**A**) Photographs of the morphology of the WT and transgenic lines (bar = 2 cm); (**B**) FA content; (**C**) CA content; (**D**) CHA content. The error bars represent the standard error (*n* = 3) (*p* < 0.05).

To investigate the accumulation of several allelopathic phenolic acids in *R. glutinosa*, we specifically chose mature root tissues (the medicinal part of the plant), which are the most sensitive tissue to replanting disease, for use in the experiments. We measured the contents of CA, FA and CHA in the roots of the selected transgenic lines and WT plants transplanted into soil (Figure 5). Overall, FA showed the highest content in all roots, followed by CA. In addition, the contents of these phenolic acids mostly showed the same trend, and the contents of all of the acids differed significantly in the roots of different plant types; i.e., the phenolic acid contents of RgC3H-OX roots were highest, followed by those of the WT (i.e., RgC3H-OX > WT > RgC3H-OX), while the lowest contents were observed in RgC3H-RNAi plants. For example, the FA contents of the roots of the *RgC3H*-overexpressing RgC3H-OX-S1 and -S3 lines were approximately 2.08- and 1.99-fold those of the WT, respectively, while the FA contents of the roots of the *RgC3H*-repression RgC3H-RNAi-S1 and -S2 lines were approximately 0.41- and 0.46-fold those of the WT, respectively. Furthermore, the CA and FA contents showed significant differences between the RgC3H-OX-S1 and RgC3H-OX-S3 transgenic overexpression lines; i.e., their contents in RgC3H-OX-S1 were significantly higher than those in RgC3H-OX-S3. At the same time, the contents of all three phenolic acids in RgC3H-RNAi-S1 roots were significantly lower than those in RgC3H-RNAi-S2. The results indicated *RgC3H* overexpression increased the production of these phenolic acids, while its repression reduced their production. Moreover, the RgC3H-OX-S1 line showed the highest accumulated levels of these phenolic acids, while the RgC3H-RNAi-S1 line showed the lowest. The RgC3H-OX-S1 and RgC3H-RNAi-S1 lines were therefore chosen for further study.

### 2.5. Analysis of the Accumulation and Release Amount of Allelopathic Phenolic Acids in Transgenic Roots

**Figure 6:**
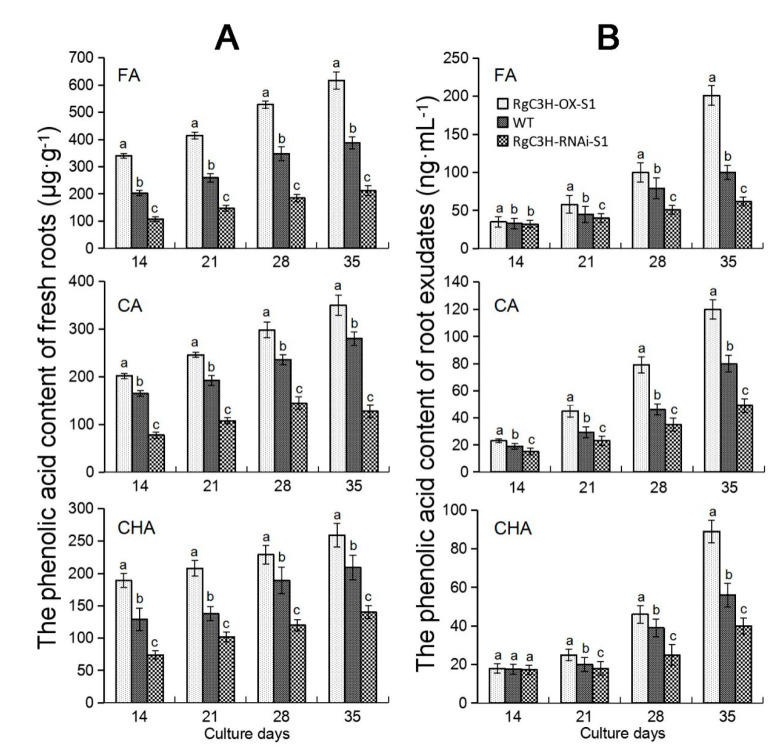
FA, CA and CHA accumulation amount of *R. glutinosa* seedling roots (**A**) and their release amount from these root exudates (**B**). The error bars represent the standard error (*n* = 3) (*p* < 0.05).

We collected RgC3H-transgenic seedling roots and their exudates under sterilized culture conditions (Figure S5) to further verify phenolic acid accumulation and test their release amount under *RgC3H* regulation. The accumulation of FA, CA and CHA mostly showed the same tendency in the roots of the three plant types (i.e., RgC3H-OX-S1, WT and RgC3H-RNAi-S1) at various stages (Figure 6A). For example, their accumulation in RgC3H-OX-S1 roots was highest, while that in RgC3H-RNAi-S1 roots was lowest. In particular, the contents of FA in RgC3H-OX-S1 roots at all examined stages were significantly higher than those in the WT, while the FA contents of RgC3H-OX-S1 roots were significantly lower than those in WT. The results verified that *RgC3H* positively altered the accumulation of allelopathic phenolic acids in phenolic acid/phenylpropanoid biosynthesis pathways. 

Similarly, in the analysis of the root exudates, we found that the accumulation of these phenolic acids in more than three stages mostly showed the same change tendency in the exudates of the roots of the three types of plants (i.e., RgC3H-OX-S1>WT>RgC3H-RNAi-S1) (Figure 6B). Moreover, following longer culture times (especially 35 days of culture), the change tendency of the accumulation of these phenolic acids became increasingly significant. For example, in the FA accumulation process determined from the various root exudates, the FA contents of the RgC3H-OX-S1 root exudates exhibited an approximately 1.29- or 2.01-fold increase relative to the WT content after culture for 21 or 35 days, respectively, while the FA contents of the root exudates of the RNAi repression line (RgC3H-RNAi-S1) at the corresponding times exhibited an approximately 0.89- or 0.62-fold decrease relative to the WT content, respectively. The results indicated that the overexpression or repression of *RgC3H* altered the accumulation and release amount of these allelopathic phenolic acids in *R. glutinosa* roots; moreover, the accumulation of these phenolic acids showed a positive relationship to their release in the roots.

### 2.6. Gene Expression Related to the Phenolic Acid/Phenylpropanoid Pathways in RgC3H-Transgenic Roots

**Figure 7:**
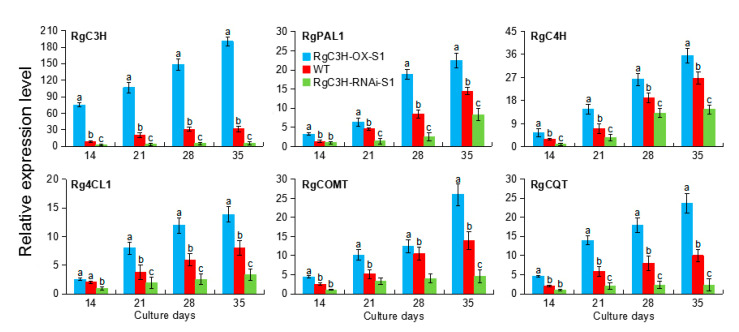
Expression patterns of the six genes involved in these allelopathic phenolic acid/phenylpropanoid biosynthesis pathways in *R. glutinosa* roots. The error bars represent the standard error (*n* = 3) (*p* < 0.05).

To gain a better understanding of the molecular regulatory mechanism of *RgC3H* in the phenolic acid/phenylpropanoid pathways, the expression of the *RgC3H*, *RgPAL1*, *RgC4H*, *Rg4CL1*, *RgCOMT,* and *RgCQT* genes was analyzed in the transgenic roots from sterile cultures after 14 to 35 days. Overall, the expression levels of the six genes in RgC3H-OX-S1 roots mainly exhibited a significant increase (1.2–6.0 folds) relative to those in the WT at these corresponding time points (especially after 35 days of culture); while their expression levels in RgC3H-RNAi-S1 roots mainly exhibited a significant decrease (0.7–0.2 fold) relative to those in the WT (Figure 7). For example, after culture for 35 days, *RgC3H* expression in RgC3H-OX-S1 and RgC3H-RNAi-S1 exhibited an approximately 6.0-fold increase and 0.17-fold decrease, respectively, relative to that in the WT, further confirming the overexpression or repression of *RgC3H* in these transgenic lines. The expression levels of the other five genes in the RgC3H-overexpressing (RgC3H-OX-S1) or RgC3H-repressioning (RgC3H-RNAi-S1) roots during most of the examined culture stages were significantly higher or lower, respectively, than those in the WT. For example, after culture for 35 days, the levels of *RgPAL1*, *Rg4CL1* and *RgCQT* expression in RgC3H-OX-S1 roots exhibited approximately 1.55-, 1.74- and 2.36- fold significant increases, respectively, relative to those in the WT; in contrast, their expression levels in RgC3H-RNAi-S1 exhibited an approximately 0.58-, 0.42- and 0.23-fold decreases relative to those in the WT. The results indicated the expression amount of these genes involved in the phenolic acid/phenylpropanoid pathways were consistently altered by the overexpression and repression of *RgC3H* genes.

## 3. Discussion

### 3.1. Characterization of the RgC3H Gene as a P450 Hydroxylase in R. glutinosa

The P450 gene family constitutes the third largest known gene family, and *Arabidopsis* possesses 245 P450 genes [23]. Only 60 of plant P450 genes have been functionally characterized; thus, more than 70% of P450 genes remain to be explored [24,25]. Studies have shown that some plant P450s participate in many phenylpropanoid pathways [15,20,25]. All of the P450 genes of *R. glutinosa* remain to be identified and they have not been functionally characterized. C3H, a member of the P450 family, has been extensively examined in some plants due to its important role in the biosynthesis of some phenolic acids in many phenylpropanoid pathways [15,17,21,26]. In this study, we successfully identified and cloned a full-length *RgC3H* cDNA from *R. glutinosa*. The RgC3H sequence is highly similar to those of other plant C3Hs; specifically, it has retained the characteristic set of conserved domains (including proline-rich region, heme-iron and heme-iron ligand signatures) and active residues and exhibits the typical characteristics of the P450 family [15], suggesting that the RgC3H may possess potential functions of cytochrome P450 proteins. In some plants, C3H is an important driver of protein-protein interaction of the phenolic acid/phenylproanoid pathways at the ER, performing the hydroxylation of diverse phenolics [15,27]. The subcellular localization of the RgC3H was identified in the ER by an experimental confirmation combined with in silico prediction, in agreement with the subcellular localization of most P450 hydrogenases in Arabidopsis, tobacco and poplar and *Artemisia annua* [27,28,29,30]. The RgC3H localization provides an important basis for the further verification of its roles in the phenolic acid/phenylproanoid pathways in *R. glutinosa*. 

### 3.2. RgC3H Overexpression and Repression Reveal a Contribution to Allelopathic Phenolic Acid Biosynthesis 

C3H is a key rate-limiting enzyme responsible for hydrogenation in the second step in the biosynthesis of some allelopathic phenolic acids (such as FA, CA and CHA) of phenylpropanoid pathways [16,21,22], which has been verified in model plant systems [15,17,31,32]. Here, to reveal the potential molecular function of the *RgC3H* in the biosynthesis of allelopathic phenolic acids, we constructed the RgC3H overexpression and RNAi repression vectors and transformed these vectors into *R. glutinosa* to generate transgenic plants. First, under field-soil-potting culture conditions, our data indicated that the overexpression of *RgC3H* in *R. glutinosa* resulted in significant increases in the FA, CA and CHA contents of the roots, whereas the repression of the *RgC3H* led to significant decreases in these phenolic acids. Thus, the overexpression or repression of *RgC3H* can alter phenolic acid accumulation in roots, suggesting that the *RgC3H* is involved in allelopathic phenolic acid biosynthesis. Second, to further confirm the involvement of the *RgC3H* in these phenolic acid biosynthesis pathways, we measured the amount of the pathways in the roots of the transgenic seedlings under in vitro sterile culture conditions, and our data showed that the overexpression or repression of the *RgC3H* mostly led to a significant increase or decrease, respectively, in the phenolic acid contents of the roots. In mung bean and coffee tree, the involvement of several C3Hs in CHA biosynthesis has be verified [19,33]. In Arabidopsis, a *C3H* lesion mutant cannot synthesize some phenolic compounds [34]. Similarly, the repression of an endogenous *C3H* transcript in hybrid poplar reduces the total cell wall lignin content and the levels of soluble phenolic products of phenylpropanoid biosynthesis [31]. Thus, we concluded that the overexpression or repression of *RgC3H* can alter the production of FA, CA and CHA in *R. glutinosa* roots, indicating that the *RgC3H* is positively involved in the corresponding biosynthetic pathways. These phenolic acids are considered to be allelopathic compounds in *R. glutinosa* [3,6,35] and some other plants [14] and are secreted from the roots and accumulate in the root rhizosphere [10,36,37], thus directly or indirectly leading to the development of replanting disease [5,38]. However, the molecular regulatory mechanism of allelopathic compound release is still unclear in *R. glutinosa* roots. Thus, the functional characterization of the role of *RgC3H* in the biosynthesis of these allelopathic phenolic acids lays a theoretical foundation for further exploring whether the *RgC3H* regulates the release of these compounds from *R. glutinosa* roots.

### 3.3. RgC3H Positively Alters the Release Amount of Allelopathic Phenolic Acids from R. glutinosa Roots Via the Activated Phenolic Acid/Phenylpropanoid Pathways

To ensure their survival, plants can generally not only synthesize secondary metabolites that accumulate in the cell but also produce and release to the environment secondary metabolites as allelopathic compounds to avoid intracellular damage caused by the excessive accumulation of these metabolites in vivo, thus changing their growth environment (including allelopathic compound concentrations, microbial communities, etc.) [10,12,37,38,39]. Although the complexity of the ecological environments of plant soils [5,39] makes it difficult to analyze root exudates, sterilized tissue culture systems can eliminate the interference caused by microorganisms and other factors in soils to facilitate the analysis of allelochemicals release amount [40]. In such a system, our results indicated that the overexpression of *RgC3H* increased allelopathic phenolic acid production, and the release of these compounds from *R. glutinosa* roots was simultaneously increased, while the repression of *RgC3H* reduced the production of these allelopathic phenolic acids and their release. Moreover, protein-protein associations are well established for some phenylpropanoid biosynthesis genes [15,27,41]. Positive linear relationships between PALs, C4Hs, 4CLs and C3Hs have been reported in Arabidopsis [41], tobacco [29], poplar [42], and petunia [15]. Our study indicated that the expression of the key genes (*RgPAL1*, *RgC4H*, *Rg4CL1*, *RgCQT*, etc.), which are involved in the phenolic acid/phenylpropanoid biosynthesis pathways [18,21], was mostly upregulated in *RgC3H*-overexpressing *R. glutinosa* roots, while they were downregulated in *RgC3H*-repressioning roots, which implied that *RgC3H* positively activates phenolic acid/phenylpropanoid metabolism networks, increasing the accumulation of some phenolic acids (e.g., CA, FA and CHA). In petunia, the RNAi repression of an endogenous C3H transcript leads to a decrease in the phenolic products of the phenylpropanoid biosynthesis, reducing the amount of the emitted floral volatiles [15]. Here, we inferred that the *RgC3H* overexpression can promote the biosynthesis of some allelopathic phenolic acids in the phenylpropanoid pathways to avoid the intracellular damage caused by their excessive accumulation in *R. glutinosa* root cells. Accordingly, some excessive phenolic acids could be released to the extracellular environment from *R. glutinosa* roots, thus increasing the phenolic acids contents of root exudates. Conversely, RgC3H repression decreased the biosynthesis of some phenolic acids in vivo, reduced their excessive accumulation in vivo and could cause less release of excessive phenolic acid contents to the extracellular environment. Thus, we considered that the *RgC3H* could positively alter the molecular networks of the phenolic acid/phenylpropanoid pathways and control the release amount of some allelopathic phenolic acids in *R. glutinosa* roots. 

The accumulation of allelopathic phenolic acids from *R. glutinosa* root exudates can damage the rhizosphere microecological environment and inhibit nutrient uptake, resulting in replanting disease [4,5,6,10,37]. Previous studies have shown that continuous monoculture induces the upregulation of these proteins (e.g., RgPAL, RgC4H, RgC3H and Rg4CL) and activates phenolic acid/phenylpropanoid pathways, possibly promoting the release of allelopathic compounds from *R. glutinosa* roots [4]. Our study also speculated that *RgC3H* activates allelopathic phenolic acid/phenylpropanoid pathways and positively controls the release of these allelochemicals to the *R. glutinosa* root extracellular environment. Since the autotoxic effect of these allelochemicals is positively correlated with their rhizosphere concentration [10], we inferred that the *RgC3H* could promote their concentration increase, likely participating in the development of replanting disease. Although the detail regarding the role of RgC3H in the molecular regulation of these phenolic acid release process still requires considerable experimental validation in *R. glutinosa*, the functional characterization of *RgC3H* and the preliminary investigation of its regulatory role in the release of the allelopathic compounds lay a theoretical foundation for revealing the molecular mechanism of replanting disease development. 

## 4. Materials and Methods

### 4.1. Cloning of the Full-Length cDNA of the RgC3H Gene 

To clone the gene sequence, *R. glutinosa* cultivar “Wen 85-5” roots were sampled at the root elongation stage (60 days after seedling emergence). Total RNA from *R. glutinosa* was isolated using TRIzol (Invitrogen, Carlsbad, USA) as recommended by the manufacturer. A Nanodrop 2000 instrument (Thermo Scientific, Wilmington, DE, USA) was used to measure the RNA concentration. A 1 μg aliquot of total RNA was reverse-transcribed into cDNA using Hiscript III Reverse Transcriptase (Vazyme, Nanjing, China) in a total volume of 20 µL using oligo(dT) primers. A putative *RgC3H* sequence was obtained from *R. glutinosa* transcriptome data [4]. Sequence analysis by using BlastN [43] indicated that it was highly homologous to C3Hs from other plant species. Specific primers (Table S3) of the *RgC3H* sequence were designed by using oligo 7.0 software, and the cDNA sequence was amplified by PCR using PrimeSTAR^®^ HS DNA Polymerase (Takara, Tokyo, Japan). The product was purified with the TaKaRa MiniBEST Agarose Gel DNA Extraction Kit and subcloned into the pMD-18 vector (Takara, Tokyo, Japan), which was then used to transform *E. coli*. All constructs were verified by sequencing (Sangon, Shanghai, China)

### 4.2. In Silico Analysis

The *RgC3H* ORF sequence was analyzed using NCBI ORFfinder [44]. The molecular weight and predicted pI of the putative RgC3H protein were obtained using the ProtParam tool (https://www.expasy.org/protparam) [45], and its secondary structure was predicted by using the SOPMA program (https://www.expasy.org/proteomics/protein_structure) [46]. The topological analysis of the transmembrane regions of RgC3H was conducted using the TMHMM-2.0 program (http://www.cbs.dtu.dk/services/TMHMM-2.0) [47], and its subcellular localization was predicted using the PSORT (https://www.psort.org) [48] and Plant-mPLoc (http://www.csbio.sjtu.edu.cn/bioinf/plant) [49] programs. Structural analysis of the deduced protein was performed using Conserved Domains (http://www.ncbi.nlm.nih.gov/Structure/cdd/wrpsb.cgi) [50] and ScanProsite (https://prosite.expasy.org) [51]. Multiple sequence alignment of C3Hs was performed using DNAMAN6.0. To illustrate the evolutionary relationships between RgC3H and some C3H sequences from other plant species reported, these C3H sequences were obtained by BlastP searches against Genbank (https://blast.ncbi.nlm.nih.gov) databases with default parameters [43]. The phylogeny of these C3H protein sequences was inferred using the neighbor-joining method implemented in the MEGA v7.0 package, applying 1000 bootstrap replicates and a Poisson correction [52]. 

### 4.3. Construction of RgC3H Vectors 

For the various *RgC3H* destination vectors, the primers for the amplification of the full ORF sequence of *RgC3H*, extending from the upstream “ATG” start codon site to the downstream region including the stop codon, or a partial 322 bp fragment of the gene were designed by using Oligo7.0 software and are shown in Table S3. The *RgC3H* sequences were amplified by PCR using PrimeSTAR^®^ HS DNA Polymerase (Takara, Tokyo, Japan). The products were purified, subcloned and sequenced as the above methods mentioned.

For the subcellular localization of *RgC3H*, the ORF of the gene sequence was inserted into the pBI121 vector (Biovector Science Lab, China) under the control of the CaMV35S promoter, fused with the N-terminus of the GFP gene, to generate the CaMV35S:GFP-RgC3H construct. For *RgC3H* overexpression in *R. glutinosa*, the full ORF of the *RgC3H* sequence was cloned into the pBI121 vector to generate the RgC3H-OX construct. For the construction of the RNA interference (RNAi) vector, the specific 322-bp sequence of *RgC3H* was used to generate an inverted repeated transgene, which was then cloned into the pRNAi-GG vector (Biovector Science Lab, China) under the control of the CaMV35S promoter to generate the RgC3H-RNAi construct. These constructs were introduced into onion or *R. glutinosa* by *Agrobacterium*-mediated transformation. 

### 4.4. Agrobacterium-Mediated Transformation 

The resulting constructs were transformed with the *Agrobacterium tumefaciens* GV3101 strain using the freeze-thaw method [53] and verified by sequencing (Sangon, Shanghai, China). 

### 4.5. Transient Expression for Subcellular Localization

For the subcellular localization of RgC3H in onion, the GV3101 strain, which was transformed into the CaMV35S:GFP-RgC3H construct (an empty vector CaMV35S:GFP as the control), was cultured (OD600  =  0.8) for collection and then infiltrated into onion epidermal cells. The transfected epidermal regions were examined after 48 h of coculture and highlighted the cellular component using ER-Tracker Red (Beyotime, C1041, China), a specific fluorescent probe for endoplasmic reticulum (ER) [54]. The transfected epidermal regions were analyzed with a fluorescence microscope (FV1000 MPE, Olympus) at an excitation wavelength of 488 nm to visualize GFP fluorescence. 

### 4.6. Establishment and Confirmation of R. glutinosa Transformation

For the *RgC3H* overexpression and repression, the sterilized leaves of *R. glutinosa* seedlings were transformed via *Agrobacterium*-mediated transformation. The leaves of *R. glutinosa* were sectioned into 5 mm^2^ pieces, and 100 leaf explants were dipped into a bacterial suspension (OD600 = 0.8) of *A. tumefaciens* GV3101, harboring the RgC3H-OX or RgC3H-RNAi transformation construct. After 30 min, the explants were blotted dry with autoclaved filter paper. The explants were placed back onto the cocultivation medium and incubated in the dark for 48 h at 26 °C. The explants were transferred to callus induction/selection medium containing solidified MS nutrient medium [55] and 30 g·L^−1^ sucrose, 0.5 mg·L^−^^1^ NAA, 1 mg·L^−1^ 6-BA, 100 mg·L^−1^ kanamycin, 200 mg·L^−1^ timentin, and 7.8 g·L^−1^ agarose at pH 5.8 [56]. The explants were transferred to fresh medium every 3 weeks. When adventitious buds developing from the calli had grown to approximately 3 cm in height, they were dissected and transferred to a new culture bottle containing shoot elongation medium consisting of MS medium, 30 g·L^−1^ sucrose, 0.5 mg·L^−1^ NAA, 3 mg·L^−1^, 100 mg·L^−1^ kanamycin, 200 mg·L^−1^ timentin, and 7.8 g·L^−1^ agarose. The shoots forming a few leaves were transferred to a rooting medium containing MS salts, 30 g sucrose, 0.05 mg·L^−1^ NAA, and 8 g agarose. The leaves of the transgenic rooted and non-transgenic seedlings (the WT, as a control) were collected at the six-leaf stage. 

To evaluate whether the overexpression and repression constructs had been integrated into the transgenic plant genome, total genomic DNA was isolated from the roots of the transgenic lines using the cetyltrimethylammonium bromide (CTAB) method [57]. We then applied the CaMV35S promoter gene-specific primers (Table S3) for amplification from isolated genomic DNA using previously published protocols [58]. 

To assess *RgC3H* transcript levels, the positive transgenic and WT seedlings were transplanted to pots containing organic matrix nutrition soils and grown for 15 days in a greenhouse that was programmed to remain at a constant temperature of 28 °C with a 14-h-light/10-h-dark cycle. The positive transgenic lines were confirmed via qRT-PCR analysis.

### 4.7. QRT-PCR Analysis 

Total RNA samples of the transgenic and WT R. *glutinosa* roots at various stages were isolated and reverse-transcribed as the mentioned above. Primer sequences (Table S4) were designed using Beacon Designer v8.01 software [59], and a *Actin* gene from *R. glutinosa* (Genebank ID: EU526396.1) was used as a reference sequence. Each 20 µL reaction contained 0.1 μM of forward and reverse primer, 10 µL AceQ® Universal SYBR qPCR Master Mix (Vazyme, Nanjing, China) and 50 ng cDNA. Negative control reactions contained no cDNA. The PCR program included an initial denaturing step (95 °C/30 s), followed by 38 cycles of 95 °C/5 s, 58 °C/10 s and 72 °C/15 s. The 2^−ΔΔ*C*t^ method [60] was used to estimate relative expression levels and the data were normalized on the basis of the reference gene expression level. QRT-PCR analysis for each sample was performed in three biological replicates.

### 4.8. Transgenic R. glutinosa Transplantation and Culture

For the measurement of CA, FA and CHA contents in *R. glutinosa* roots, the positive transgenic and WT seedlings cultured in nutrition soils were transplanted to pots in *R. glutinosa* field soils and grown in the above mentioned greenhouse. The roots of the transplanted plants in the field soils were collected at mature stages (150 days after transplantation).

To further measure the phenolic acid contents of the *R. glutinosa* roots and their exudates and the root expression profiles of genes related to the phenolic acid/phenylpropanoid pathways, sterilized transgenic *R. glutinosa* seedlings were raised until the six-leaf stage. The plants were subsequently transferred to fresh tubes containing 40 mL of the same nutrient medium under the sterilized culture conditions described above. Measurements of these roots and their exudates were performed after 14, 21, 28, and 35 days. Each sample was represented by three replicate seedlings.

### 4.9. Phenolic Acid Content Assay

To estimate the phenolic acid contents of root samples, the tissue was ground, extracted in 5 mL of 1 M NaOH per g of root tissue, and passed through a 0.22 µm filter. The pH of the filtrate was adjusted to 2.5 with HCl, and the filtrate was extracted five times in an equal volume of ethyl acetate. The pooled extracts were lyophilized, and the residue was dissolved in 5 mL of methanol for the measurement of FA CA and CHA content via HPLC analysis. The extraction of the root exudates followed the same procedure used for the measurement of these phenolic acids.

For HPLC analysis of each example, CA, FA and CHA were quantified using a 1260 HPLC device (Agilent Technologies, Santa Clara, CA, USA) equipped with a ZORBAX Eclipse XDB-C18 column (4.6 × 250 mm, 5 μm). The mobile phases for CA, FA and CHA were a 1:1 mixture of 100% methanol and 0.2 M phosphoric acid, a 1:1 mixture of 100% methanol and 0.1 M phosphoric acid, and a 1:1 mixture of 100% acetonitrile and 0.05 M phosphoric acid, respectively, provided at a constant flow of 11 mL min^−1^. The detection of CA, FA and CHA was performed at 323, 300 and 327 nm, respectively, and the column temperature was maintained at 30 °C. All experiments were performed in triplicate.

### 4.10. Statistical Analyses

Data were analyzed by one-way analysis of variance (ANOVA) [61]. The subsequent multiple comparisons were tested based on the least significant difference (LSD) and Duncan’s multiple range test. Different significance was set at *p* < 0.05.

## 5. Conclusions

The *RgC3H* participates in allelopathic phenolic acid biosynthesis and positively alters their production and release amount from *R. glutinosa* roots via the activated phenolic acid/phenylpropanoid pathways. The presumption is that RgC3H contributed to the accumulation of allelochemicals in *R. glutinosa* rhizosphere, possibly affecting the replanting disease development.

## Supplementary Materials

The following are available online at https://www.mdpi.com/2223-7747/9/5/567/s1, Figure S1: The phenolic aicd biosynthesis by C3H catalyation in the phenylpropanoid pathways in some plants, Figure S2: Topological analysis of RgC3H using the TMHMM2 program, Figure S3: The constructs for the RgC3H subcellular localization (**a**), overexpression (**b**) and repression (**c**), Figure S4: The generation of *RgC3H* transgenic *R. glutinosa* plants, Figure S5: Part of RgC3H transgenic and WT *R. glutinosa* seedlings under sterilized culture conditions at various stages, Table S1: Characteristics of the deduced translation products of the RgC3H cDNA, Table S2: Identity of RgC3H and other plant C3Hs, Table S3: The primers of RgC3H used to clone, vector constructs, and the screening of positive transgenic lines, Table S4: Primer sequences used for the qRT-PCR analysis of these genes.
