# Supplementary material for: RgC3H Involves in the Biosynthesis of Allelopathic Phenolic Acids and Alters Their Release Amount in Rehmannia glutinosa Roots
Source: Plants (Basel). 2020 Apr 29;9(5):567. doi: 10.3390/plants9050567 (PMC7284580; doi:10.3390/plants9050567)
Supplement: Supplementary file 1 [file plants-09-00567-s001.zip › Supplementary files - plants-790409/Supplementary file 1.docx]

**PAL**

**COMT CQT**

**C3H 4CL**

*p*-coumaroyl-CoA

**C3H**

**TAL**

Phenylalanine

Tyrosine

Transcinnamic acid

Caffeic aid

Ferulic acid

*p*-coumaroyl-quininc acid

Chlorogenic acid

Their derivatives and other phenol compounds

*p*-Coumarate

**Figure S1.** The phenolic aicd biosynthesis by C3H catalyation in the phenylpropanoid pathways in some plants. Note: TAL= tyrosine ammonia-lyase, PAL= phenylalanine ammonia-lyase, C4H= cinnamate-4-hydroxylase, C3H= *p* -coumarate-3-hydroxylase, COMT=caffeic acid O-methyltransferase, 4CL= 4-coumarate-CoA ligase, CQT= hydroxycinnamoyl-CoA quinate hydroxycinnamoyl transferase.


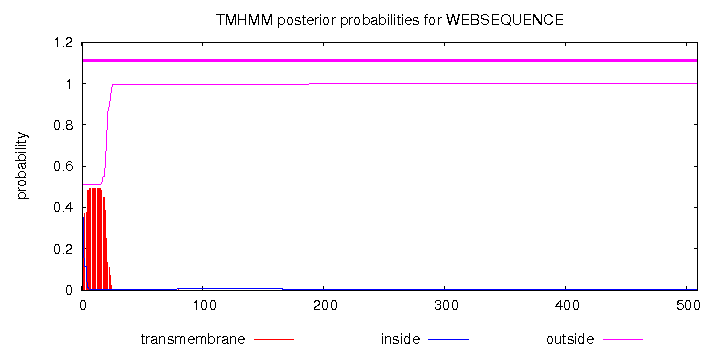


**Figure S2.** Topological analysis of RgC3H using the TMHMM2 program.


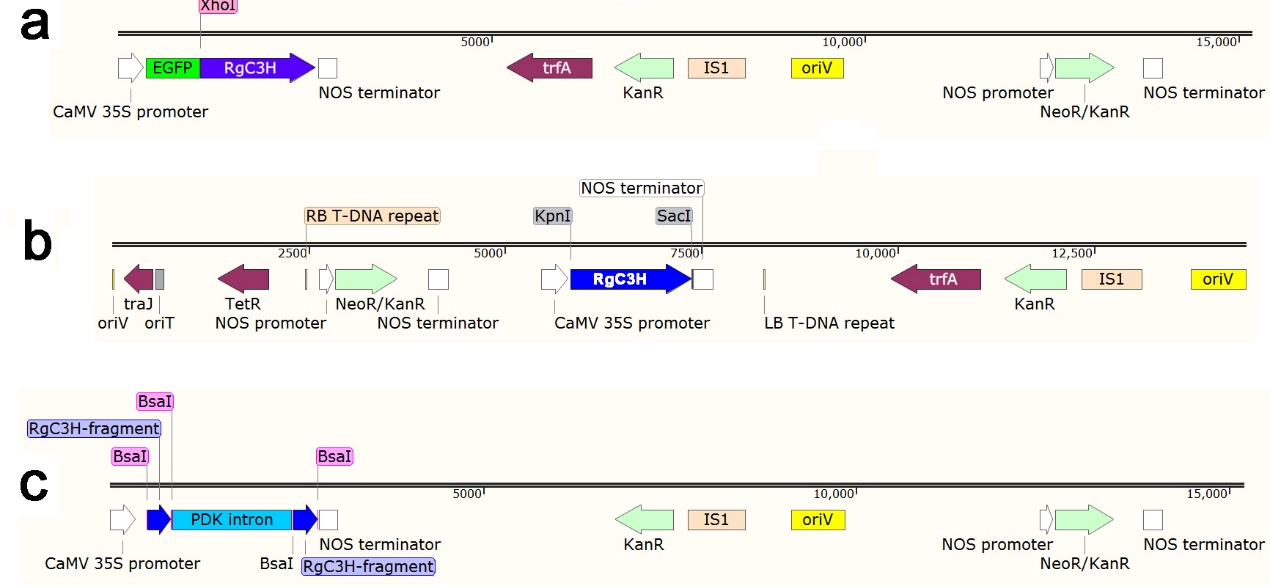


**Figure S3.** The constructs for the RgC3H subcellular localization (**a**), overexpression (**b**) and repression (**c**).


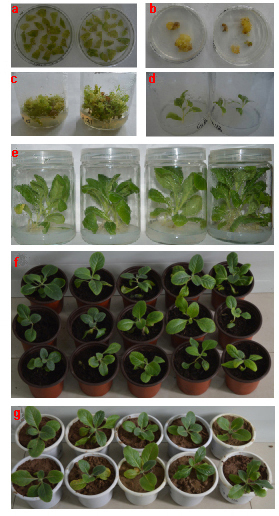


**Figure S4.** The generation of *RgC3H* transgenic *R.* *glutinosa* plants. (**a**) Co-cultivation of the explants and *Agrobacterium tumefaciens*; (**b**) The callus induction/selection medium from the explants-transferred; (**c**) The adventitious buds induction/selection medium from the callus; (**d**) The shoots were induced and rooted plants in selection medium; (**e**) The transgenic seedlings were [generate](C:/Program%20Files%20(x86)/Youdao/Dict/8.5.1.0/resultui/html/index.html#/javascript:;)d in the selection medium; (**f**) These seedlings-transplanted to pots in the organic matrix nutrition soils for adapting soil environments; (**g**) These seedling-transplanted to pots in field soils.


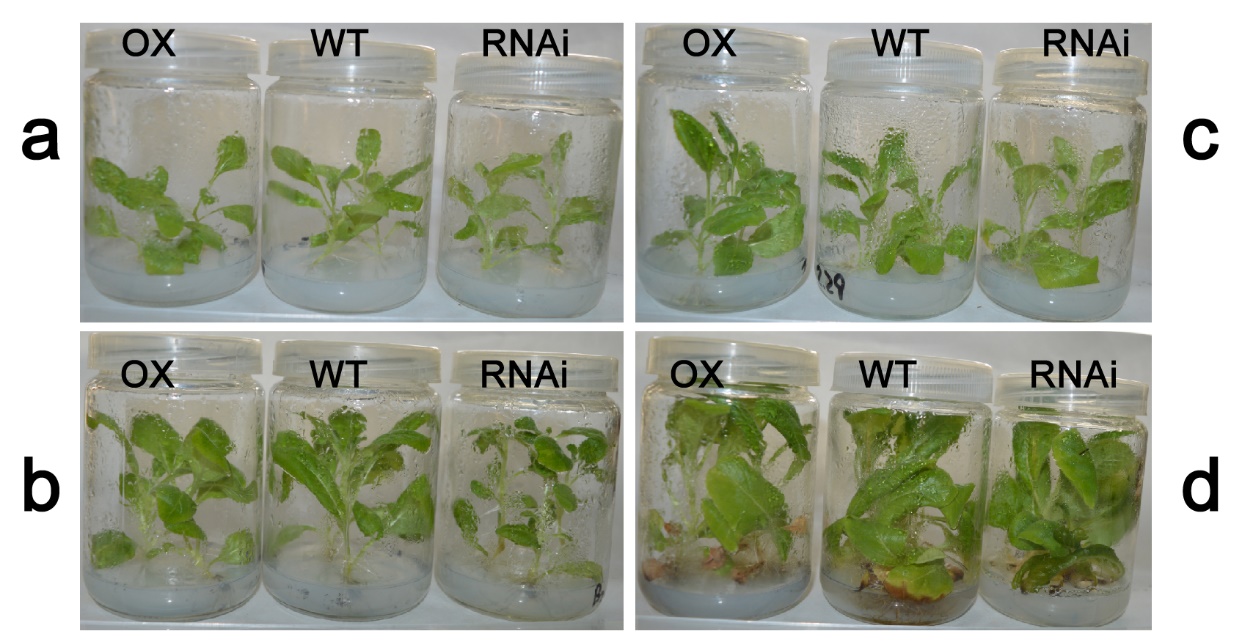


**Figure S5.** Part of RgC3H transgenic and WT *R.* *glutinosa* seedlings under sterilized culture conditions at various stages. (**a**) 14 days after culture; (**b**) 21 days after culture; (**c**) 28 days after culture; (**d**) 35 days after culture.
