# Supplementary material for: RgC3H Involves in the Biosynthesis of Allelopathic Phenolic Acids and Alters Their Release Amount in Rehmannia glutinosa Roots
Source: Plants (Basel). 2020 Apr 29;9(5):567. doi: 10.3390/plants9050567 (PMC7284580; doi:10.3390/plants9050567)
Supplement: Supplementary file 1 [file plants-09-00567-s001.zip › Supplementary files - plants-790409/Supplementary file 2.docx]

**Table S1.** Characteristics of the deduced translation products of the RgC3H cDNA.

| **Program Type** | **Characteristics** | **Results** |
| --- | --- | --- |
| ProtParam | Number of amino acids | 509 |
|  | Formula | C2608H4136N706O727S28 |
|  | Molecular weight | 57911.5 |
|  | Theoretical pI | 8.89 |
|  | Instability index | 31.11 |
|  | Aliphatic index | 90.22 |
|  | Grand average of hydropathicity (GRAVY) | -0.22 |
| SOPMA | Alpha helix of the secondary structure | 51.87% |
|  | Extended strand of the secondary structure | 9.43% |
|  | Beta turn of the secondary structure | 6.88% |
|  | Random coil of the secondary structure | 31.83% |
| TMHMM2 | Transmembrane segment | 1 |
| PSORT | Subcellular localization | Endoplasmic reticulum |
| Plant-mPLoc |  |  |

**Table S2.** Identity of RgC3H and other plant C3Hs.

| **Name** | **Accession No.** | **Plant Species** | **Identity (%)** |
| --- | --- | --- | --- |
| NpC3H | AXU39897.1 | *Narcissus papyraceus* | 64.19 |
| NtC3H | AGI97941.1 | [*Narcissus tazetta*](https://blast.ncbi.nlm.nih.gov/Blast.cgi) | 63.60 |
| BeC3H | AFD29885.1 | *Bambusa emeiensis* | 61.67 |
| SbC3H | BAJ09387.1 | *Scutellaria baicalensis* | 86.47 |
| CfC3H | AYK02678.1 | *Catalpa fargesii* | 94.70 |
| ShC3H | AIA24412.1 | *Sinopodophyllum hexandrum* | 65.69 |
| PaC3H | CAK22403.1 | *Picea abies* | 41.05 |
| GbC3H | AAY54293.1 | *Ginkgo biloba* | 63.33 |
| ClC3H | AFX98060.1 | *Cunninghamia lanceolata* | 63.14 |
| AtC3H | NP_850337.1 | *Arabidopsis thaliana* | 70.78 |
| FtC3H | AHA14499.1 | *Fagopyrum tataricum* | 66.60 |
| EsC3H | AIS92508.1 | *Epimedium sagittatum* | 70.14 |
| PtC3H | ABY85195.1 | *Populus alba x Populus grandidentata* | 69.55 |
| HcC3H | AGA60530.1 | *Hibiscus cannabinus* | 69.93 |
| GsC3H | ALH21661.1 | *Gossypium hirsutum subsp. hirsutum* | 66.67 |
| AkC3H | AOX49220.1 | *Acacia koa* | 67.19 |
| VuC3H | XP_027930224.1 | *Vigna unguiculata* | 67.65 |
| SiC3H | XP_011091866.1 | *Sesamum indicum* | 66.99 |
| PxhC3H | AVA30528.1 | *Petunia x hybrida* | 65.64 |
| WsC3H | ADM47799.1 | *Withania somnifera* | 67.19 |
| CaC3H | NP_001311496.1 | *Capsicum annuum* | 66.41 |

**Table S3.** The primers of *RgC3H* used to clone, vector constructs, and the screening of positive transgenic lines.

| **Use** | **Primer** | **Sequence (5'to 3')** | **Tm (°C)** |
| --- | --- | --- | --- |
| Cloning the complete sequence | Forward | CACAGCGAAAGTCAACAATGGCTATC | 61.1 |
| if | Reverse | CCAAGCGTTTAAGGGATATATTTTGCAACAG | 61.0 |
| CaMV35S:GFP-RgC3H construct | Forward | ATGGCTATCCCTCTCCTCATTCTCTC | 59.9 |
|  | Reverse | GTCGAAGAACTCATACATTTCCAACA | 58.8 |
| RgC3H-OX construct | Forward | ATGGCTATCCCTCTCCTCATTCTCTC | 59.9 |
|  | Reverse | GTCGAAGAACTCATACATTTCCAACA | 58.8 |
| RgC3H-RNAi construct | Forward | TAAGTTGGGTACCAAGAAGTCCTTCGC | 64.5 |
|  | Reverse | TCTCGGGTTTCGTACCATCTCGGC | 64.3 |
| Amplify *CaMV35S* gene amplify | Forward | GAGACTTTTCAACAAAGGGTAATATCC | 57.2 |
|  | Reverse | GTATGAAGATGAACAAAGCCCTGAA | 57.0 |

**Table S4.** Primer sequences used for the qRT-PCR analysis of these genes.

| **Gene Name** | **Primer** | **Sequence (5' to 3')** | **Tm (°C)** |
| --- | --- | --- | --- |
| RgC3H | Forward | ATTAGAGAAGATGAAGTTAC | 56.3 |
| if | Reverse | TTGTTATGTGAAGGAATG | 56.5 |
| RgPAL1 | Forward | TTAAGGCTGCTCAGAAGATG | 58.5 |
|  | Reverse | TGTGGCGATGTCCTAAGA | 58.8 |
| RgC4H1 | Forward | TAGGCAGTTATGACATTC | 56.3 |
|  | Reverse | AGTGTGAAGGATTGTTAG | 56.2 |
| Rg4CL1 | Forward | ATGATTGGAGTGATGAAC | 56.6 |
|  | Reverse | AACATTGACCACATTCTT | 56.7 |
| RgCOMT | Forward | ATTCACTTCAATCCAACA | 56.3 |
|  | Reverse | CAACATTAGAGCAGAGAT | 56.4 |
| RgCQT | Forward | ACGGAGAGGAATGATACA | 58.1 |
|  | Reverse | GACAAGTGGGATAATACCTAT | 58.3 |
| RgActin | Forward | GCCATGTATGTTGCTATT | 57.5 |
|  | Reverse | CACCAGAATCCATCACTA | 57.4 |
